# Loss of function in *rpms-1* does not enhance phenotypes of *rpm-1* mutants

**DOI:** 10.17912/micropub.biology.001396

**Published:** 2024-12-05

**Authors:** Yue Sun, Daniela Gaio, Bokun Xie, Kentaro Noma, Zilu Wu, Yishi Jin

**Affiliations:** 1 University of California, San Diego, La Jolla, California, United States

## Abstract

The *C. elegans* E3 ubiquitin ligase RPM-1 consists of 3,766 amino acids, with a RING finger domain at the C-terminus that functions to target the DLK-1 kinase for degradation for synapse development and axon termination. *rpms-1 (* for *rpm-1 short,* aka F07B7.12 *)* resides 35 kb away from *rpm-1* on chromosome V, and is a near-perfect 12 kb duplication of *rpm-1 ,* including the entire promoter region and coding sequences. RPMS-1 consists of 1,964 amino acids and is identical to the N-terminal half of RPM-1 , except the last 40 amino acids. Previous studies showed that transgenic overexpression of the duplicated region of *rpm-1 (+)* did not rescue synapse defects of *rpm-1* loss of function mutants. Here, using CRISPR editing, we generated a double knockout of *rpm-1* and *rpms-1* . We find that axon and synapse defects in *rpm-1 rpms-1* double mutants resemble those in *rpm-1* single mutants. Expression levels of endogenously tagged DLK-1 protein are increased to a comparable degree in *rpm-1* and *rpm-1 rpms-1* mutants, compared to the control. These data, along with previous transgene expression analysis, support the idea that *rpms-1* does not have a major role in RPM-1-mediated cellular processes.

**Figure 1. Double mutants of rpm-1 rpms-1 resemble rpm-1 single mutants:**
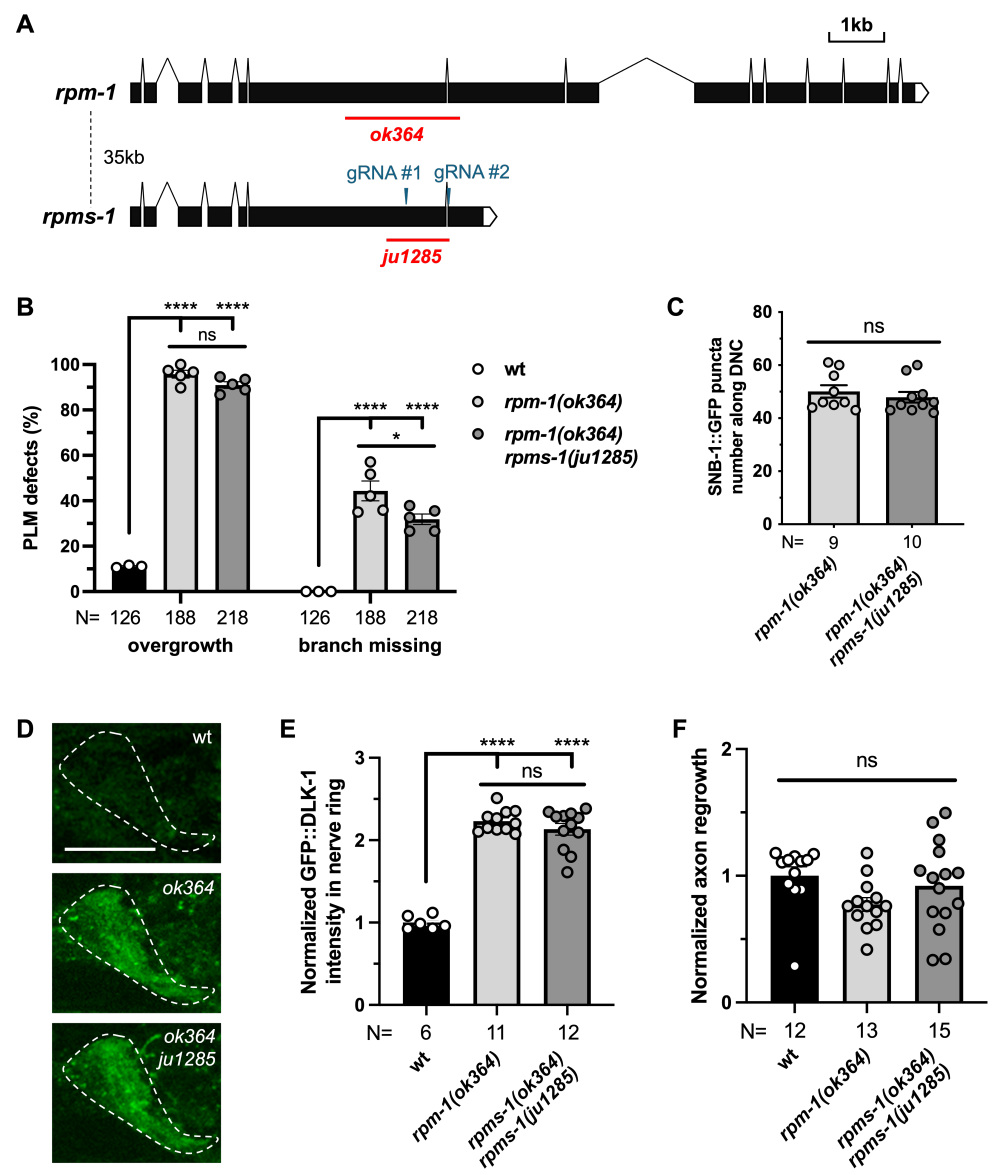
A. Schematics of *rpm-1* and *rpms-1* gene model. Black boxes represent exons. Blue arrowheads point at gRNA targeting sites for CRISPR-Cas9 genome editing. The deletion alleles *ok364* and *ju1285* result in truncated proteins due to frameshift followed by premature stop codons. B. Quantification of axon termination defects and absence of synapse branch of PLM neuron visualized by *muIs32 ( mec-7 p::GFP)* . Numbers of animals analyzed are shown below the bar graphs. Statistics: one-way ANOVA test for multiple comparison corrected with the false discovery rate (FDR) method of Benjamini and Hochberg. Error bars: SEM. ****p<0.0001, *0.01<p<0.05, ns=p>0.05. C. Quantification of the total number of synaptic puncta of GABAergic neurons in the dorsal nerve cord (DNC) visualized by *juIs1 (unc-25p::SNB-1::GFP)* . Numbers of animals analyzed are shown in the bar graphs. Statistics: unpaired t test. Error bars: SEM. ns=p>0.05. D. Representative images of knock in GFP:: DLK-1 ( *ju1579* ) in the nerve ring region. White dashes outline the region of interest (ROI) for quantification in E. Scale bar: 20 µm. E. Quantification of the fluorescence intensity of GFP:: DLK-1 ( *ju1579* ) normalized to the average value of wildtype (wt) animals. Numbers of animals analyzed are shown below the bar graphs. Statistics: one-way ANOVA test for multiple comparison corrected with the false discovery rate (FDR) method of Benjamini and Hochberg. Error bars: SEM. ****p<0.0001, ns=p>0.05. F. Quantification of PLM axon regrowth length after laser axotomy, normalized to the average value of wildtype (wt) animals. Numbers of animals analyzed are shown below the bar graphs. Statistics: one-way ANOVA test for multiple comparison corrected with the false discovery rate (FDR) method of Benjamini and Hochberg. Error bars: SEM. ns=p>0.05.

## Description

*rpm-1* encodes an E3 ubiquitin ligase of 3766 amino acids, containing multiple domains that include an RCC1 like domain at the N-terimus, PHR domains in the middle, and a RING domain and a zinc finger box domain at the C-terminus (Schaefer et al., 2000; Zhen et al., 2000) . Loss of function of RPM-1 causes abnormal presynaptic development in several types of neurons, including the GABAergic motor neurons (Zhen et al., 2000) and touch receptor neurons (Schaefer et al., 2000) , as well as overextension of touch neuron axons (Grill et al., 2007) . During the cloning of *rpm-1* (Schaefer et al., 2000; Zhen et al., 2000) , it was discovered that *F07B7.12* , located at 35 kb away from *rpm-1* ( **Figure** **1A** ), is a near-perfect genomic duplication of 12 kb of *rpm-1* from the promoter region to coding sequences for ~1900 amino acids, hence named as *rpms-1* ( *rpm-1* s hort). A transgene expressing the duplicated region of *rpm-1* in an *rpm-1* null mutant did not rescue synapse defects, showing that *rpms-1* cannot compensate for the function of *rpm-1* (Zhen et al., 2000) . To address the role of *rpms-1* in *rpm-1-* mediated function directly, we used CRISPR editing to generate a double knockout of *rpm-1* and *rpms-1 .*

As *rpm-1* and *rpms-1* have nearly identical sequences, sgRNAs designed for *rpms-1* also bind *rpm-1* and result in similar genome editing. We thus took advantage of *rpm-1 ( ok364 )* animals, which have 2,221-bp deletion, removing part of large exon 6 and exon 7 and causing protein truncation due to frameshift. We designed two sgRNAs within the *ok364* deletion region. Using the co-CRISPR strategy (Friedland et al., 2013) , we generated a new allele *ju1285* , which is a 1,218-bp deletion in exon 6 of *rpms-1* and causes frameshift followed by a premature stop codon. The *rpm-1 ( ok364 ) rpms-1 ( ju1285 )* animals are homozygous viable, grossly indistinguishable from *rpm-1 ( ok364 ).* We examined touch receptor neuron morphology and GABAergic neuron synapses, and found no significant differences in axon overextension or synaptic puncta, with double mutants showing a slight reduction in touch neuron synapse branches ( **Figure** **1B, 1C** ). DLK-1 is negatively regulated by RPM-1 via protein degradation (Nakata et al., 2005) . We measured the fluorescence intensity of endogenously expressed GFP:: DLK-1 ( *ju1579* ) as previously described (Sun and Jin, 2023) , and did not detect a significant difference between the single and double mutant ( **Figure** **1D, 1E** ). Moreover, after axon injury by laser axotomy, both *rpm-1* and *rpm-1 rpms-1* mutants showed axon regrowth to a similar level, comparable to that of control ( **Figure** **1F** ). Together, these analyses show that *rpms-1* does not contribute to *rpm-1* mediated regulation of neuronal development.

## Methods

1. CRISPR-Cas9 mediated genome editing

We used CRISPR sgRNA design tool http://crispr.mit.edu (Feng Zhang's lab). Mutagenesis primers were ordered (EtonBio) and used to insert sgRNAs into *eft-3 p* ::cas9-NLS-pU6 empty vectors by Phusion PCR. Plasmids were treated with DpnI to digest plasmids of bacteria origin (methylated). DH5α transformation followed. The following plasmids were obtained and sequenced: pCZ890 (sgRNA#1) and pKEN268 (sgRNA#2). CZ3007 *rpm-1 ( ok364 )* worms were injected with 50 ng/μl of each plasmid DNA and 5 ng/μl pCFJ104 *myo-3 p* ::RFP co-injection marker. F1 positive worms were let lay enough eggs and genotyped with primers YJ10660 (5'-ACCGATATGACTGGATATGAAAATCGTC) and YJ11134 (5'-GGCCATTCGCTCCCATAAC) for *rpms-1 ( ju1285 ).*

2. Laser axotomy

We cut PLM axons in anesthetized L4 worms using a near-infrared Ti-Sapphire laser (KMLabs) as described (Wu et al., 2007) . We used *muIs32* ( *mec-7 p* ::GFP) to visualize touch neuron morphology and axon regeneration. To anesthetize worms for surgery and imaging, we put animals in the agar pad and used 0.1~1% 1-phenoxy-2-propanol in M9 buffer. For confocal imaging, we used an LSM710 confocal microscope to take Z-stack images of live anesthetized worms. Representative images for axon regrowth are projections or single layers of Z-stack images. Regrowth lengths were measured from maximum transparency projections of one single z-stack using Zeiss AIM software.

3. Fluorescence microscopy and GFP intensity measurement

Confocal images of the nerve ring region were collected from L4 immobilized in 2 mM levamisole (Sigma) in M9 buffer using a Zeiss LSM710 confocal microscope. Projections of Z-stack images (1 μm/section) are shown in the figure. GFP intensity from the region of interest (ROI) was analyzed in ImageJ, and the graph was generated in GraphPad Prism.

4. Statistics

For comparisons involving multiple groups, we used one-way ANOVA in GraphPad Prism. When comparing two groups, we used an unpaired t test with two-tailed P value.

## Reagents

CZ3007 : *juIs1* [ *unc-25p::* SNB-1::GFP]; *rpm-1 ( ok364 )* V

CZ9840 : *muIs32* [ *mec-7 p* ::GFP] II; *rpm-1 ( ok364 )* V

CZ10969 : *muIs32* [ *mec-7* p::GFP] II

CZ22189 : *juIs1* [ *unc-25p::* SNB-1::GFP]; *rpm-1 ( ok364 ) rpms-1 ( ju1285 )* V

CZ22190 : *rpm-1 ( ok364 ) rpms-1 ( ju1285 )* V

CZ22191 : *muIs32* ; *rpm-1 ( ok364 ) rpms-1 ( ju1285 )* V

CZ26773 : *muIs32* [ *mec-7 p* ::GFP] II; *rpm-1 ( ok364 )* V

CZ26774 : *muIs32* [ *mec-7 p* ::GFP] II; *rpm-1 ( ok364 ) rpms-1 ( ju1285 )* V

CZ25941 : GFP:: *dlk-1 ( ju1579 )* I

CZ26775 : GFP:: *dlk-1 ( ju1579 )* I; *rpm-1 ( ok364 ) rpms-1 ( ju1285 )* V

CZ26787 : GFP:: *dlk-1 ( ju1579 )* I; *rpm-1 ( ok364 )* V
